# CT protocol optimisation in PET/CT: a systematic review

**DOI:** 10.1186/s40658-020-00287-x

**Published:** 2020-03-16

**Authors:** V. Bertolini, A. Palmieri, M. C. Bassi, M. Bertolini, V. Trojani, V. Piccagli, F. Fioroni, S. Cavuto, M. Guberti, A. Versari, S. Cola

**Affiliations:** 1 Medical Physics Unit, Azienda USL-IRCCS, Reggio Emilia, Italy; 2 Nuclear Medicine Unit, Azienda USL-IRCCS, Reggio Emilia, Italy; 3 Medical Library, Azienda USL-IRCCS, Reggio Emilia, Italy; 4 Medical Physics Specialization School, Università degli Studi di Bologna, Bologna, Italy; 5 Research and Statistics Infrastructure, Azienda USL-IRCCS, Reggio Emilia, Italy; 6 Health Care Professionals Unit, Azienda USL-IRCCS, Reggio Emilia, Italy

**Keywords:** PET/CT, Computed tomography, CT protocol optimisation

## Abstract

**Purpose:**

Currently, no consistent guidelines for CT scans used within PET/CT examinations are available. This systematic review provides an up-to-date overview of studies to answer the following questions: What are the specific CT protocols used in PET/CT? What are the possible purposes of requiring a CT study within a PET/CT scan? Is the CT protocol obtained from a dosimetric optimisation study?

**Materials and method:**

PubMed/MEDLINE, Cochrane Library, Embase and Scopus were systematically searched for relevant studies in accordance with the PRISMA statement. The literature search was conducted from January 2007 until June 2019. Data derived from studies were standardized in order to reduce possible biases, and they were divided into clinically homogeneous subgroups (adult, child or phantom). Subsequently, we divided the CT protocol intents into 3 types (anatomic localization only, attenuation correction only and diagnostic purpose). A narrative approach was used to summarise datasets and to investigate their heterogeneity (due to medical prescription methodology) and their combination in multiseries CT protocols. When weighted computed tomography dose index (CTDI_w_) was available, we calculated the volumetric computed tomography dose index (CTDI_vol_) using the pitch value to make the results uniform. Eventually, the correlation between protocol intents and CTDI_vol_ values was obtained using a Kruskal–Wallis one-way ANOVA statistical test.

**Result:**

Starting from a total of 1440 retrieved records, twenty-four studies were eligible for inclusion in addition to two large multicentric works that we used to compare the results. We analyzed 87 CT protocols. There was a considerable range of variation in the acquisition parameters: tube current–time product revealed to have the most variable range, which was 10–300 mAs for adults and 10–80 mAs for paediatric patients. Seventy percent of datasets presented scans acquired with tube current modulation, 9% used fixed tube current and in 21% of them, this information was not available. Dependence between mean CTDI_vol_ values and protocol intent was statistically significant (*p* = 0.002). As expected, in diagnostic protocols, there was a statistically significant difference between CTDI_vol_ values of with and without contrast acquisitions (11.68 mGy vs 7.99 mGy, *p* = 0.009). In 13 out of 87 studies, the optimisation aim was not reported; in 2 papers, a clinical protocol was used; and in 11 works, a dose optimisation protocol was applied.

**Conclusions:**

According to this review, the dose optimisation in PET/CT exams depends heavily on the correct implementation of the CT protocol. In addition to this, considering the latest technology advances (i.e. iterative algorithms development), we suggest a periodic quality control audit to stay updated on new clinical utility modalities and to achieve a shared standardisation of clinical protocols. In conclusion, this study pointed out the necessity to better identify the specific CT protocol use within PET/CT scans, taking into account the continuous development of new technologies.

## Introduction

In the last few years, there has been a growing interest in using CT scans into PET/CT examinations. PET/CT scanners play an important role in modern nuclear medicine as a result of their hybrid nature, which combines metabolic imaging (PET) with a morphological one offered by the computed tomography (CT). The increasing number of commercially available PET tracers and a robust scientific literature have confirmed the PET/CT leading role in diagnostic and therapeutic care, in particular for oncological diseases [1, 2]. Some examples from the wide range of possible PET/CT applications are staging of disease, early (“interim”) response, late response to therapy, restaging and follow-up. In addition to the most well-known applications in the field of oncology (56%), the use of PET/CT exams must also be considered in several other fields, such as the study of dementias and temporal epilepsies (32%) and the absolute quantification of coronary flow in cardiology (12%) [3]. Quite recently, other so-called *“emerging”* PET/CT applications have gained considerable attention, e.g. the study of phlogosis, infections, fever of undetermined origin (FUO), large vessel vasculitis (LVV) and endocarditis [4, 5]. Therefore, the optimisation of acquisition protocols is required, due to these increasing PET/CT applications. This is an important issue for patients who must repeat the PET/CT examination several times due to their care pathway; thus, a periodic analysis and update of the acquisition protocols is necessary to reduce the “cumulative” dose to the patient. This subject is handled following the most recent Council of the European Union Reference Directive (2013/59/EURATOM) [6]. In fact, in article 56, subparagraph 4 concerning *Optimisation,* it is stated that “the optimisation includes the selection of equipment, the consistent production of adequate diagnostic information or therapeutic outcomes, the practical aspects of medical radiological procedures, quality assurance, and the assessment and evaluation of patient doses or the verification of administered activities, taking into account economic and societal factors”. Fahey et al. [7] argued that consistent guidelines do not exist for the acquisition of a CT scan as part of a paediatric PET/CT examination.

In clinical studies, optimisation of acquisition protocols ensures a safer and more efficient examination with the same high clinical information. The correct radiopharmaceutical dose required to perform a good-practice PET scan is currently described by several national and international publications and guidelines written by scientific associations [8, 9]. Conversely, CT protocols used in PET/CT and their contribution to dosimetry are not supported by a likewise robust scientific literature [3, 8, 9]. Within a PET/CT examination, the possible CT acquisitions are made for a diagnostic purpose, anatomic localization of PET tracer uptake or only for attenuation correction of PET images. The diagnostic use (with or without contrast enhancement) is required for a more precise disease assessment and it normally needs a higher dose compared to the others. On the other hand, if the CT scan is performed only for anatomic localization, the acquisition technique can be optimised to reduce the patient dose of about 50–80%, if compared to the diagnostic reference levels [10]. Finally, if the attenuation correction of PET images is required, the CT scan needs to be taken to generate a low-resolution attenuation map. In this latter case, the image quality is worse (i.e. noisier) than the diagnostic one as a result of the CT image processing needed to match the PET resolution. In this case, it is easy to reach a reduction in CT doses from 10 to 100 times compared to the diagnostic reference levels [10]. The choice of the CT intent and therefore the associated acquisition protocol is the first step towards the dose optimisation aim which, as it is well known, depends on the acquisition parameters, i.e. tube current-time product, tube voltage, pitch, slice thickness, rotation time and collimation [1]. We conducted a systematic review to further elucidate on this topic and to explore how the studies dealt with the need of optimised acquisition CT protocols in PET/CT scans while maintaining a reasonably high diagnostic image quality when it is required. Our systematic review focuses on all available publications about the different applications of CT scans during a PET/CT acquisition. This review aims to answer the following questions: (1) What are the specific CT protocols used in PET/CT? (2) What are the possible purposes of requiring a CT study within a PET/CT scan? (3) Is the CT protocol obtained from a dosimetric optimisation work?

## Materials and methods

The review protocol was registered at Prospero International prospective register of systematic reviews (CRD42019118076).

### Eligibility criteria

This systematic review followed the recommendations of the PRISMA-P (Preferred Reporting Items for Systematic Review and Meta-Analysis Protocols) 2015. We considered as a participant (**P**), a population composed of adult patients, paediatrics patients, phantoms and/or a combination of those two categories, as intervention (**I**), the technical acquisition parameters of a CT study in a PET/CT exam and as an outcome (**O**), the identification of a dedicated or optimised CT acquisition protocol, which was classified by type of patient, site of disease and protocol intent. The inclusion criteria of the records in the review process are the presence of a specific CT protocol in PET/CT exams or a dosimetric CT optimisation.

### Search strategy

A comprehensive literature search was performed using four electronic databases: PubMed, Cochrane Library, Embase and Scopus. Since PET/CT scans are relatively recent imaging devices in clinical Nuclear Medicine, the literature search was conducted from January 2007 until January 2020. Mesh terms and free text were modelled in search strategies for databases using the one designed for PubMed: “Protocol * AND (reduction OR optimisation OR limit) AND (CT OR Computed tomography) AND (positron emission tomography OR PET)”. All duplicate publications have been excluded. The search was restricted to English language studies only with no limits for publication status. The authors did not contact other institutions or authors in order to identify further studies.

### Data extraction

Three review authors independently selected records by reading their titles and abstracts and extract general study characteristics (the title of the study, name of the first author, year of publication, journal, abstract, corresponding reviewer and research keys) for records that matched the inclusion criteria, using a customised data extraction form. Authors’ decisions were compared, and disagreement was reassessed until consensus was reached. After excluding records that lacked information on CT parameters, any drafted studies with full text were included in the review process, while conference proceedings and monographs were discarded.

At this stage, further articles that did not meet the inclusion criteria at the full-text reading were identified and excluded.

Full-text articles have been selected by reviewers in duplicate; any doubt was resolved by including another author’s opinion in the selection process. From each selected full-text article, the following parameters were identified and extracted: population, type of scan, specific anatomical district, purpose of CT execution, eventual use of contrast agent, type of tomography used, CT scan parameters (kV, mAs, Pitch, AEC, etc.), image quality (if indicated), comparison with standard CT protocols in PET/CT exams and its result and a reference bibliography.

No contact with the authors of the records for complementary information was necessary.

### Risk of bias

No quality scoring for study selection was applied. Articles were read by reviewers in duplicate, using the third reviewer only in cases of doubt. Data derived from studies were standardized in order to reduce possible biases. Data extracted from studies that did not meet the criteria for their possible standardization were excluded.

### Data synthesis

The data were divided into clinically homogeneous subgroups (adult, child or phantom); subsequently, each subgroup was written in alphabetical order by a manufacturer name. The use of phantoms is preliminary and indispensable in all those works that aim at the dosimetric optimisation of the acquisition clinical protocols. Indeed, the correct phantoms choice enables the dosimetric impact simulation of ionizing radiation exposure in predefined body segments. All available protocol technical data, protocol intents and scan districts were reported whenever possible.

We examined datasets reporting the protocol intent distribution to investigate their heterogeneity due to medical prescription methodology using a narrative approach.

We divided the CT protocol intents into 3 types (anatomic localization only, attenuation correction only and diagnostic purpose) and their combination in multiseries CT protocols. The CT protocol intent distribution was performed using percentage.

When weighted computed tomography dose index (CTDI_w_) was available, we calculated CTDI_vol_ dividing the CTDI_w_ by the respective pitch value to make the results uniform (whenever possible, otherwise, we left “CTDI_w_” indicated). Data were summarised as mean ± standard deviation (SD). The correlation between protocol intents and CTDI_vol_ values was obtained using a Kruskal–Wallis one-way ANOVA statistical test. A *p* value less than 0.05 was set to consider a correlation as statistically significant.

We investigated the scan district for every protocol intent, counting the number of protocols and taking into account when the x-ray contrast agent was used. Furthermore, we examined the presence of an optimisation aim by counting the number of works describing a clinical protocol dose optimisation instead of a “simple” clinical protocol description.

When a datum was not declared, we used N.A. as an abbreviation.

### Meta-bias(es)

We registered under the same manufacturer name that all of the equipment models made by the same manufacturer.

As stated before, not all papers had a CT dose index (CTDI_w_ or CTDI_vol_) and/or protocol intent reported.

The patient number was not always available; thus, the results do not take into account this datum. Besides, for clinical protocols where CTDIs were measured on a phantom, the patients’ number and the CTDI error (here intended as SD) could not have been reported.

## Results

### Literature search

The computer-aided search revealed 2516 records from PubMed, Cochrane Library, Embase and Scopus (Table 1). After removing duplicates, 1440 records remained.

**Table 1:** Database distribution of found records

Database (***n***° of record)				***n***° of total records (with duplicates)	***n***° of total records (without duplicates)
Cochrane	Scopus	EMBASE	Medline		
320	368	1,203	625	2,516	1,440

**Fig. 1:**
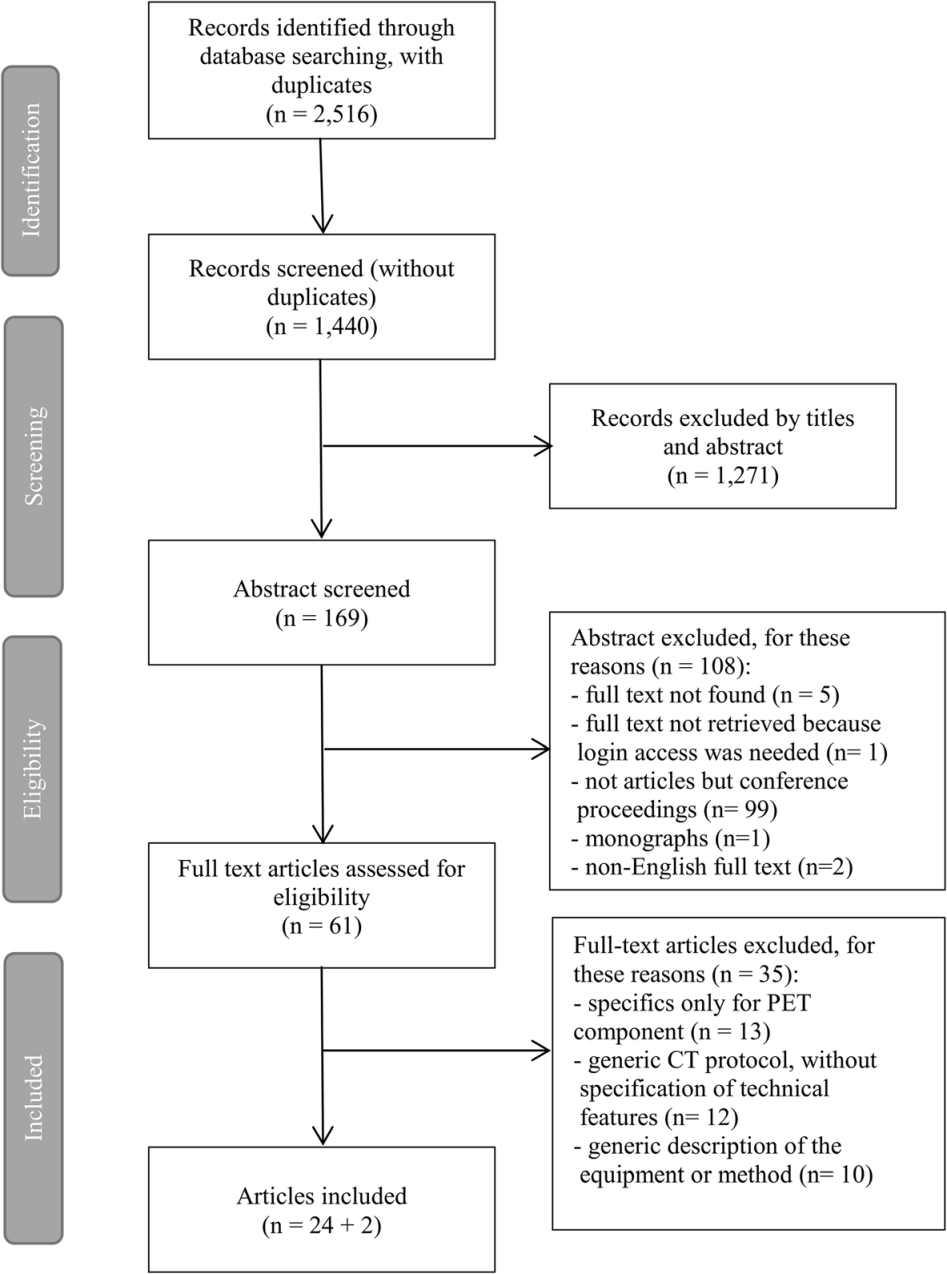
PRISMA-P flow chart: articles selection process

After excluding records without information on CT acquisition parameters, 169 abstracts were screened (Fig. 1).

Non-English full-texts, monographs and conference proceedings were excluded. Five full-text articles were not found, and one full-text required login access. Thus, 61 full-text articles were assessed by the reviewers for eligibility. Further, 35 articles were removed from the database due to one of the following reasons: specifics only for the PET component or generic CT protocol without specification of technical features and generic description of the equipment or method. After this final selection, 26 articles remained. Among these, 2 were large multicentre studies, and we used them to discuss our results. We investigated whether the dose results obtained in those multicentre studies were in accordance with our review.

The included articles were divided into studies related to adults (Table 2), paediatric patients (Table 3) and phantoms (Table 4). Each table shows results in alphabetical order of manufacturers and describes the number of articles, the number of datasets, the authors, the number of patients (if available) and the CT acquisition parameters. There was a considerable range of variation in the acquisition parameters: tube current–time product revealed the most variable range which was 10–300 mAs. In adult patients, the most found tube voltage was 120 kV (60%), with a range of 110–140 kV. Eighty-eight percent of datasets used a helical CT, 6% axial CT, 2% step and shoot CT and 4% N.A. Fifty percent of datasets presented scans acquired with tube current modulation, 10% with fixed tube current and 40% N.A. Similar acquisition parameter variability was also reported in paediatric studies. Tube current–time product ranged between 10 mAs and 80 mAs, while the most frequent tube voltage was 120 kV, ranging in an interval between 80 kV and 140 kV. Seventy percent of datasets presented scans acquired with tube current modulation, 9% with fixed tube current and it was not indicated in 21% of them.

**Table 2:** Eligible adult studies

Manufacturer	Number of articles	Number of datasets	Author	No. of pts	Protocol parameters												Dose index	
					Scan type	Protocol intent	Scan district	X-Ray contrast use	kVp	mAs modulation	mA or mAs (reference)	Other current selection	Rotation time (s)	Slice thickness (mm)	Pitch	Collimation	CTDI_vol_ (mGy)	ED (mSv)
GE Medical System	10	15	Sera et al. [11]	N.A.^a^	axial	C	WB	N	120	N.A.	80 mAs	N	0.8	N.A.	N	25	10.86	N.A.
			Goldberg et al. [12]	20	helical	B	WB + Pelvis	Y	120	Y	100–300 mAs	N	0.8	3.75	N.A.	N.A.	N.A.	N.A.
			Sonoda et al. [13]	100^b^		B	WB	N	140	N	80 mA	120 mA (if weight > 100 kg)	0.8	N.A.	1.5	4 × 25	N.A.	10.6 (± 2.2)
			Lautamaki et al. [14]	92		B	Heart	N	140	Y	50–100 mAs	N	N.A.	N.A.	N.A.	N.A.	N.A.	N.A.
			Liu, et al. [15]	14^b^		A	WB	N	120	Y	N.A.	N	0.5	N.A.	0.98	N.A.	N.A.	male 24.73 ± 8.54 female 18.68 ± 3.35
				14^b^		D	WB + Abdomen	N	120	Y	N.A.	N	0.5	N.A.	0.98	N.A.	N.A.	male 21.20 ± 8.94 female 14.79 ± 3.35
				14^b^		D	WB + Abdomen	Y	120	Y	N.A.	N	0.5	N.A.	0.98	N.A.	N.A.	male 57.13 ± 17.88
			Brix et al. [16]	N.A.^a^		C	WB	Y	140	N.A.	150 mAs	N	N.A.	2.5	1.5	N.A.	14.1	19.4
			Sera et al. [11]	N.A.^a^		C	WB	N	120	N.A.	100 mAs	N	0.5	N.A.	0.82	40	6.62	N.A.
			Tonkopi et al. [17]	140		A	WB	N	120	Y	10–210 mAs	Noise index = 25	0.8	3.75	1.75	16 × 0.625	6.4 ± 2.4	8.1 ± 3.3
				100		A	WB	N	120	Y	10–210 mAs	Noise index = 27.1	0.5	3.75	1.35	16 × 1.25	4.3 ± 1.6	5.5 ± 2.1
			Son et al. [18]	69		C	Head and neck	Y	120	N	100 mA	N	0.5	3.75	N.A.	N.A.	N.A.	N.A.
			Javadi et al. [19]	15^b^		B	Heart	Y	120	Y	400–800 mA (both unenhanced and contrast medium)	N	0.35	N.A.	0.2–0.24	N.A.	N.A.	23.8 ± 4
			Javadi et al. [19]	15^b^	step and shoot	B	Heart	Y	120	Y	500 mA (unenhanced) + 600–800 mA (contrast medium)	N	0.35	2.5(unenhanced) + 0.625(contrast medium)	increment 35 mm	40	N.A.	8.8 ± 1.5
			Veronesi, et al. [20]	157	N.A	B	WB	N	140	N	80 mA	N	N.A.	N.A.	N.A.	N.A.	N.A.	N.A.
Philips Healthcare	5	17	Murray et al. [21]	20^a^	axial	B	WB	N	120	N	50 mAs	N	N.A.	5	N	N.A.	N.A.	N.A.
			Kwee et al. [22]	8	helical	B	WB	N	120	N	50 mA	N	0.75	5	N.A.	N.A.	N.A.	N.A.
			Brix et al. [16]	N.A.^a^		C	WB	Y	120	N.A.	195 mAs	N	N.A.	5	1.5	N.A.	9.5	14.2
				N.A.^a^		B	WB	N	120	N.A.	60 mAs	N	N.A.	6.5	1.5	N.A.	2.9	4.6
			Sera et al. [11]	N.A.^a^		C	WB	N	120	N.A.	71 mAs	N	0.8	N.A.	0.8	3	11.7	N.A.
				N.A.^a^		C	WB	N	110	N.A.	50 mAs	N	0.6	N.A.	1.5	5	3.67	N.A.
				N.A.^a^		C	WB	N	130	N.A.	70 mAs	N	0.6	N.A.	1.5	5	7.37	N.A.
				N.A.^a^		C	WB	N	130	N.A.	50 mAs	N	0.8	N.A.	1.5	1.25	6.24	N.A.
				N.A.^a^		C	WB	N	130	N.A.	80 mAs	N	0.6	N.A.	1.5	3	8.52	N.A.
			Saade et al. [23]	1028^b^		N.A.	WB	N	120	Y	120 mAs	N	0.5	5	0.8	N.A.	6.68 ± 1.97	N.A.
				1028^b^		N.A.	WB	N	120	Y	120 mAs	N	0.5	5	0.8	N.A.	7.29 ± 1.69	N.A.
				1028^b^		N.A.	WB	N	120	Y	120 mAs	N	0.5	5	0.8	N.A.	7.45 ± 1.59	N.A.
				1028^b^		N.A.	WB	N	120	Y	120 mAs	N	0.5	5	0.8	N.A.	9.36 ± 1.62	N.A.
				1028^b^		N.A.	WB	N	140	Y	35 mAs	N	0.75	5	0.8	N.A.	3.21 ± 0.72	N.A.
				1028^b^		N.A.	WB	N	140	Y	50 mAs	N	0.75	5	0.8	N.A.	4.4 ± 0.48	N.A.
				1028^b^		N.A.	WB	N	140	Y	65 mAs	N	0.75	5	0.8	N.A.	5.42 ± 0.44	N.A.
				1028^b^		N.A.	WB	N	140	Y	100 mAs	N	0.75	5	0.8	N.A.	7.02 ± 2.99	N.A.
Siemens Healthcare	8	18	Sera et al. [11]	N.A.^a^	axial	C	WB	N	120	N.A.	80 mAs	N	0.5	N.A.	N	5	8.09	N.A.
			Willowson, et al. [24]	483	helical	A	WB	N	120	Y	80 mAs	N	0.33	1.2	0.8	N.A.	N.A.	8.2 (3.4–23.4)
			Eiber et al. [25]	94^b^		C	WB	Y	120	Y	240 mAs	N	0.5	N.A.	N.A.	64 × 0.6	N.A.	13.84 (8.62–23.26)
				94^b^		B	Chest	N	120	Y	25 mAs	N	0.5	N.A.	N.A.	64 × 0.6	N.A.	13.84 (8.62–23.26)
			Prieto et al. [26]	322^b^		A	WB	N	120	Y	120 mAs	N	0.5	N.A.	1	16 × 1.2	5.9 ± 1.5	9.5 ± 2.8
				322^b^		A	WB	N	120	Y	100 mAs	N	0.5	N.A.	1	16 × 1.2	5.1 ± 1.3	8.0 ±5.3
				322^b^		A	WB	N	120	Y	80 mAs	N	0.5	N.A.	1	16 × 1.2	4 ± 0.8	6.2 ± 1.5
			Brix et al. [16]	N.A.^a^		C	WB	Y	130	N.A.	111 mAs	N	N.A.	4	1	N.A.	11.9	17.8
				N.A.^a^		B	WB	N	110	N.A.	30 mAs	N	N.A.	4	2	N.A.	1	1.5
				N.A.^a^		C	WB	Y	120	N.A.	200 mAs	N	N.A.	1.5	1.25	N.A.	11.2	14.3
				N.A.^a^		B	WB	N	120	N.A.	32.5 mAs	N	N.A.	0.75	1.25	N.A.	2	2.6
			Sera et al. [11]	N.A.^a^		C	WB	N	130	N.A.	70 mAs	N	0.6	N.A.	1.5	5	10.41	N.A.
				N.A.^a^		C	WB	N	130	N.A.	91 mAs	N	0.6	N.A.	1.5	5	8.88	N.A.
				N.A.^a^		C	WB	N	130	N.A.	86 mAs	N	0.6	N.A.	1.5	5	8.61	N.A.
				N.A.^a^		C	WB	N	130	N.A.	50 mAs	N	0.8	N.A.	0.8	3	4.93	N.A.
			Ciappuccini et al. [27]	21		B	WB + Head and neck	N	130	N.A.	100 mAs	N	N.A.	2.5	1	N.A.	N.A.	N.A.
			Sawicki et al. [28]	28		N.A.	WB	Y	120	Y	210 mAs	N	N.A.	5	0.8	128 × 0.6	N.A.	N.A.
			Menezes et al. [29]	237	N.A	B	WB + Liver	N	110 or 130	Y	N.A.	N	0.6	5	N.A.	9.6	N.A.	N.A.

*A* CT acquisition for anatomic localization and attenuation correction,*, B* CT acquisition for attenuation correction only (ultra-low dose), *C* CT acquisition for diagnostic purposes and attenuation correction, *D* WB CT acquisition for anatomic localization and attenuation correction + CT segmentary acquisition for diagnostic purpose, *N.A.* data not available, *WB* whole body, *Y* yes*, N* no, *CTDI* computed tomography dose index, *ED* effective dose

^a^Clinical protocol studied on phantom, reported here with the same values both in adult and phantom tables

^b^Total not differentiated

**Table 3:** Eligible paediatric studies

Manufacturer	Number of articles	Number of datasets	Author	No. of pts	Protocol parameters												Dose index	
					Scan type	Protocol intent	Scan district	X-Ray contrast use	kVp	mAs modulation	mA or mAs (reference)	Weight-based current selection	Rotation time (s)	Slice thickness (mm)	Pitch	Collimation	CTDI_vol_ (mGy)	ED (mSv)
GE Medical System	4	18	Sera et al. [11]	N.A.^c^	axial	C	WB	N	120	N	80 mAs	N	1	N.A.	N	25	5.49	N.A.
			Sonoda, et al. [13]	100^b^	helical	B	WB	N	140	N	80 mA	120 mA (if weight > 100 kg)	0.8	N.A.	1.5	4 × 2.5	N.A.	10.6 (± 2.2)
			Brady et al. [30]	N.A.^c^		B	WB	N	80	Y	65–130 mA	between 0 and 9.4 Kg	N.A.	3.75	0.985	N.A.	N.A.	N.A.
				N.A.^c^		B	WB	N	100	Y	80–160 mA	between 9.5 and 18.4 Kg	N.A.	3.75	0.985	N.A.	N.A.	N.A.
				N.A.^c^		B	WB	N	100	Y	110–210 mA	between 18.5 and 31.4 Kg	N.A.	3.75	0.985	N.A.	N.A.	N.A.
				N.A.^c^		B	WB	N	100	Y	110–220 mA	between 31.5 and 55 Kg	N.A.	3.75	0.985	N.A.	N.A.	N.A.
				N.A^c^		B	WB	N	120	Y	150–210 mA	greater than 55 Kg	N.A.	3.75	0.985	N.A.	N.A.	N.A.
			Alessio et al. [31]	N.A.		A	WB	N	120	Y	10 mAs	between 6 and 7.4 Kg	0.5	2.5	0.98	N.A.	N.A.	3.1
				N.A.		A	WB	N	120	Y	10 mAs	between 7.5 and 9.4 Kg	0.5	2.5	0.98	N.A.	N.A.	2.9
				N.A.		A	WB	N	120	Y	15 mAs	between 9.5 and 11.4 Kg	0.5	2.5	0.98	N.A.	N.A.	4.1
				N.A.		A	WB	N	120	Y	20 mAs	between 11.5 and 14.4 Kg	0.5	2.5	0.98	N.A.	N.A.	5.2
				N.A.		A	WB	N	120	Y	20 mAs	between 14.5 and 18.4 Kg	0.5	2.5	0.98	N.A.	N.A.	5
				N.A.		A	WB	N	120	Y	20 mAs	between 18.5 and 22.4 Kg	0.5	2.5	0.98	N.A.	N.A.	4.7
				N.A.		A	WB	N	120	Y	25 mAs	between 22.5 and 31.4 Kg	0.5	2.5	0.98	N.A.	N.A.	5.5
				N.A.		A	WB	N	120	Y	30 mAs	between 31.5 and 40.5 Kg	0.5	2.5	0.98	N.A.	N.A.	6.3
				N.A.		A	WB	N	120	Y	30mAs	between 40.5 and 55 Kg	0.5	2.5	0.98	N.A.	N.A.	5.6
				N.A.		A	WB	N	120	Y	35 mAs	between 55 and 70 Kg	0.5	2.5	0.98	N.A.	N.A.	5.9
				N.A.		A	WB	N	120	Y	40 mAs	greater than 70 Kg	0.5	2.5	0.98	N.A.	N.A.	5.9
Philips Healthcare	1	2	–	–	axial	–	–	–	–	–	–	–	–	–	–	–	–	–
			Sera et al. [11]	N.A.^c^	helical	C	WB	N	110	N.A.	50 mAs	N	0.6	N.A.	1.5	5	7.8	N.A.
				N.A.^c^		C	WB	N	130	N.A.	47 mAs	N	0.8	N.A.	0.8	3	5.54	N.A.
			–	–	N.A.	–	–	–	–	–	–	–	–	–	–	–	–	–
Siemens Healthcare	1	3	Sera et al. [11]	N.A.^c^	axial	C	WB	N	120	N.A.	80 mAs	N	1	N.A.	N	10	7.34	N.A.
			Sera et al. [11]	N.A.^c^	helical	C	WB	N	130	N.A.	50 mAs	N	0.8	N.A.	0.8	3	11.49	N.A.
				N.A.^c^		C	WB	N	130	N.A.	50 mAs	N	1	N.A.	0.55	3	10.92	N.A.
			–	–	N.A.	–	–	–	–	–	–	–	–	–	–	–	–	–

*A* CT acquisition for anatomic localization and attenuation correction*, B* CT acquisition for attenuation correction only (ultra-low dose), *C* CT acquisition for diagnostic purposes and attenuation correction, *D* WB CT acquisition for anatomic localization and attenuation correction + CT segmentary acquisition for diagnostic purpose, *N.A.* data not available, *WB* whole body, *Y* yes*, N* no, *CTDI* computed tomography dose index, *ED* effective dose

^b^Total not differentiated

^c^Clinical protocol studied on phantom, reported here with the same values both in paediatric and phantom tables

**Table 4:** Eligible phantom studies

Manufacturer	Number of articles	Number of datasets	Study	No. of phantom study		Protocol parameters											Dose index	
						Scan type	Protocol intent	Scan district	kVp	mAs modulation	mA or mAs (reference)	Current selection weight based	Rotation time (s)	Slice thickness (mm)	Pitch	Collimation	CTDI_vol_ (mGy)	ED (mSv)
GE Medical System	7	19	Sera et al. [11]	N.A.^a^		axial	C	WB	120	N.A.	80 mAs	N	0.8	N.A.	N	25	10.86	N.A.
			Sera et al. [11]	N.A.^c^			C	Head and neck	120	N.A.	80 mAs	N	1	N.A.	N	25	5.49	N.A.
			Huang et al. [32]	N.A.		helical	N.A.	WB	120	Y	100–300 mA	N	0.5	0.625	0.984	N.A.	N.A.	7.2 mSv female 7.42 mSv male
				N.A.			N.A.	WB	120	N	250 mAs	N	0.5	0.625	0.984	N.A.	N.A.	18.56 mSv female 18.57 mSv male
				N.A.			N.A.	WB	140	Y	150–350 mA	N	0.5	0.625	0.984	N.A.	N.A.	25.68 mSv female 25.95 mSv male
			Javadi et al. [19]	N.A.			B	Heart	120	N	600 mA	N	0.35	0.625	N.A.	N.A.	15.6	19.6
			Brady et al. [30]	N.A.^c^			B	WB	80	Y	65–130 mA	N	N.A.	3.75	0.985	N.A.	N.A.	N.A.
				N.A.^c^			B	WB	100	Y	80–160 mA	N	N.A.	3.75	0.985	N.A.	N.A.	N.A.
				N.A.^c^			B	WB	100	Y	110–210 mA	N	N.A.	3.75	0.985	N.A.	N.A.	N.A.
				N.A.^c^			B	WB	100	Y	110–220 mA	N	N.A.	3.75	0.985	N.A.	N.A.	N.A.
				N.A.^c^			B	WB	120	Y	150–210 mA	N	N.A.	3.75	0.985	N.A.	N.A.	N.A.
			Brix et al. [16]	N.A.^a^			C	WB	140	N.A.	150 mAs	N	N.A.	2.5	1.5	N.A.	14.1	N.A.
			Sera et al. [11]	N.A.^a^			N.A.	WB	120	N.A.	100 mAs	N	0.5	N.A.	0.82	40	6.62	N.A.
			Alessio et al. [31]	N.A.			A	WB	80	N	10 mAs	N	0.5	2.5	0.98	N.A.	N.A.	N.A.
				N.A.			A	WB	100	N	20 mAs	N	0.5	2.5	0.98	N.A.	N.A.	N.A.
				N.A.			A	WB	120	N	40 mAs	N	0.5	2.5	0.98	N.A.	N.A.	N.A.
				N.A.			A	WB	140	N	80mAs	N	0.5	2.5	0.98	N.A.	N.A.	N.A.
			Umeda et al. [33]	N.A.			N.A.	WB	120	Y	N.A.	N	0.5	3.75	0.938	N.A.	N.A.	N.A.
			Javadi et al. [19]	N.A.		step and shoot	D	Heart	120	N	600 mA	N.A.	0.35	0.625	N	N.A.	4.5	5.4
Philips Healthcare	3	10	Murray et al. [21]	N.A.^a^		axial	B	WB	120	N	50 mAs	N	N.A.	5	N.A.	N.A.	N.A.	N.A.
			Sera et al. [11]	N.A.^a^		helical	C	WB	120	N.A.	71 mAs	N	0.8	N.A.	0.8	3	11.7	N.A.
				N.A.^a^			C	WB	110	N.A.	50 mAs	N	0.6	N.A.	1.5	5	3.67	N.A.
				N.A.^a^			C	WB	130	N.A.	70 mAs	N	0.6	N.A.	1.5	5	7.37	N.A.
				N.A.^a^			C	WB	130	N.A.	50 mAs	N	0.8	N.A.	1.5	1.25	6.24	N.A.
				N.A.^a^			C	WB	130	N.A.	80 mAs	N	0.6	N.A.	1.5	3	8.52	N.A.
			Sera et al. [11]	N.A.^c^			N.A.	Head and neck	110	N.A.	50 mAs	N	0.6	N.A.	1.5	5	7.8	N.A.
				N.A.^c^			N.A.	Head and neck	130	N.A.	47 mAs	N	0.8	N.A.	0.8	3	5.54	N.A.
			Brix et al. [16]	N.A.^a^			C	WB	120	N.A.	195 mAs	N	N.A.	5	1.5	N.A.	9.5	N.A.
				N.A.^a^			B	WB	120	N.A.	60 mAs	N	N.A.	6.5	1.5	N.A.	2.9	N.A.
Siemens Healthcare	3	19	Sera et al. [11]	N.A.^a^		axial	C	WB	120	N.A.	80 mAs	N	0.5	N.A.	N	5	8.09	N.A.
				N.A.^c^			C	Head and neck	120	N.A.	80 mAs	N	1	N.A.	N	10	7.34	N.A.
			Kumar et al. [34]	42^b^		helical	B	N.A.	130	N	16 mAs	N	N.A.	5	N.A.	4	CTDI_w_ 5.41	N.A.
				42^b^			B	N.A.	130	N	25 mAs	N	N.A.	5	N.A.	4	CTDI_w_ 4.9	N.A.
				42^b^			B	N.A.	130	N	30 mAs	N	N.A.	5	N.A.	4	CTDI_w_ 4.32	N.A.
				42^b^			B	N.A.	130	N	35 mAs	N	N.A.	5	N.A.	4	CTDI_w_ 3.8	N.A.
				42^b^			B	N.A.	130	N	40 mAs	N	N.A.	5	N.A.	4	CTDI_w_ 3.28	N.A.
				42^b^			B	N.A.	130	N	45 mAs	N	N.A.	5	N.A.	4	CTDI_w_ 2.71	N.A.
				42^b^			B	N.A.	130	N	50 mAs	N	N.A.	5	N.A.	4	CTDI_w_ 1.73	N.A.
			Sera et al. [11]	N.A.^a^			C	WB	130	N.A.	70 mAs	N	0.6	N.A.	1.5	5	10.41	N.A.
				N.A.^a^			C	WB	130	N.A.	91 mAs	N	0.6	N.A.	1.5	5	8.88	N.A.
				N.A.^a^			C	WB	130	N.A.	86 mAs	N	0.6	N.A.	1.5	5	8.61	N.A.
				N.A.^a^			C	WB	130	N.A.	50 mAs	N	0.8	N.A.	0.8	3	4.93	N.A.
				N.A.^c^			C	Head and neck	130	N.A.	50 mAs	N	0.8	N.A.	0.8	3	11.49	N.A.
				N.A.^c^			C	Head and neck	130	N.A.	50 mAs	N	1	N.A.	0.55	3	10.92	N.A.
			Brix et al. [16]	N.A.^a^			C	WB	130	N.A.	111 mAs	N	N.A.	4	1	N.A.	11.9	N.A.
				N.A.^a^			B	WB	110	N.A.	30 mAs	N	N.A.	4	2	N.A.	1	N.A.
				N.A.^a^			C	WB	120	N.A.	200 mAs	N	N.A.	1.5	1.25	N.A.	11.2	N.A.
				N.A.^a^			B	WB	120	N.A.	32.5 mAs	N	N.A.	0.75	1.25	N.A.	2	N.A.

*A* CT acquisition for anatomic localization and attenuation correction,*, B* CT acquisition for attenuation correction only (ultra-low dose), *C* CT acquisition for diagnostic purposes and attenuation correction, *D* WB CT acquisition for anatomic localization and attenuation correction + CT segmentary acquisition for diagnostic purpose, *N.A.* data not available, *WB* whole body, *Y* yes*, N* no, *CTDI* computed tomography dose index, *ED* effective dose

^a^Clinical protocol studied on phantom, here are reported with the same values both in adult and phantom tables

^b^Total not differentiated

^c^Clinical protocol studied on phantom, reported here with the same values both in paediatric and phantom tables

### CT protocol intents used in PET/CT

According to all the 27 considered studies, a CT scan in PET/CT exams can be done for different purposes. In Table 5, we identified and reported 5 classes of protocol intents: (A) CT acquisition for anatomic localization and attenuation correction, (B) CT acquisition for attenuation correction only (ULTRA-low dose), (C) CT acquisition for diagnostic purpose and attenuation correction, (D) whole body (WB) CT acquisition for anatomic localization and attenuation correction + CT segmentary acquisition for diagnostic purpose and finally (N.A.), when data were not available.

**Table 5:** Results of protocol intent distribution

Protocol intent	No.	Percentage
A	22	25.3%
B	26	29.9%
C	24	27.6%
D	2	2.3%
N.A.	13	14.9%
Total	87	100%

*A* CT acquisition for anatomic localization and attenuation correction, *B* CT acquisition for attenuation correction only (ULTRA-low dose), *C* CT acquisition for diagnostic purpose and attenuation correction, *D* WB CT acquisition for anatomic localization and attenuation correction + CT segmentary acquisition for diagnostic purpose, *N.A.* data not available

Data analysis indicated that class B was the most represented (29.9%), followed by C, A and D (27.6%, 25.3% and 2.3% respectively). Protocol intent was not available in 14.9% of papers.

**Fig. 2:**
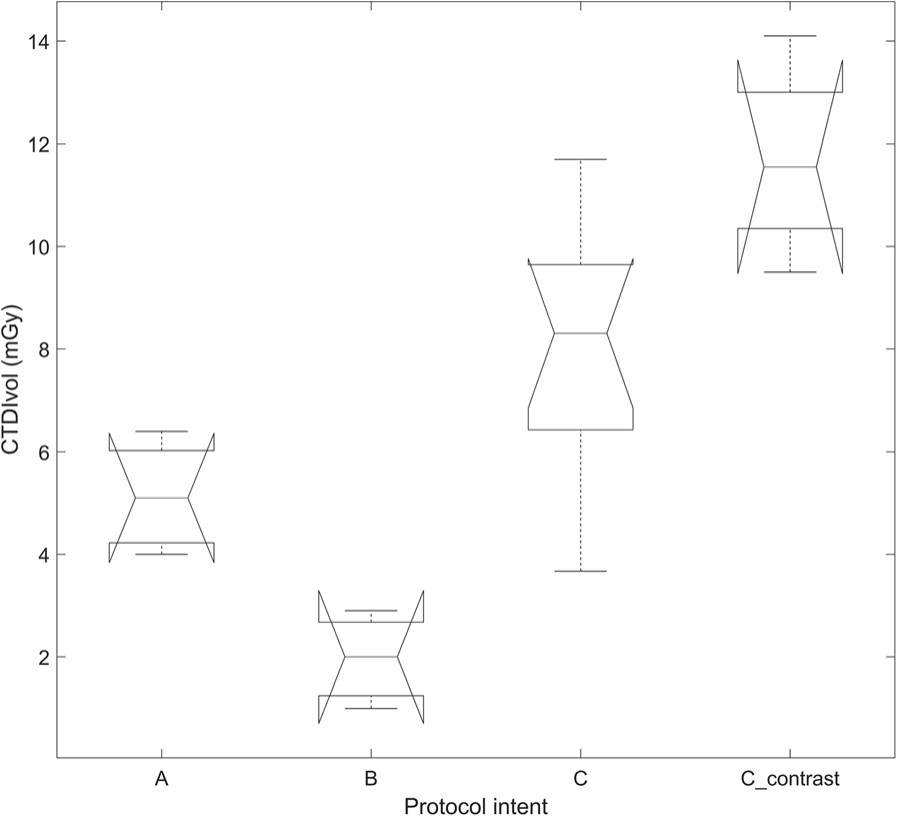
Distributions of CTDI_vol_ values for each protocol intent. **a** CT acquisition for anatomic localization and attenuation correction. **b** CT acquisition for attenuation correction only (ULTRA-low dose). **c** CT acquisition for diagnostic purposes and attenuation correction without contrast and C_contrast CT acquisition for diagnostic purposes and attenuation correction with contrast. In particular, only protocol intent C included also data about the CTDI_vol_ values of the x-ray contrast medium examinations. The error bars of protocol intent C are wider than the A and B protocols due to their intrinsic diagnostic nature. CTDI_vol_ values for protocol intents A and B were statistically different from protocol intent C (*p* = 0.002); in addition, CTDI_vol_ values were statistically different for protocol intents C and C_contrast (*p* = 0.009)

From Table 2, we calculated the mean CTDI_vol_ for each protocol intents. Only in records under protocol intent A, B and C, CTDI_vol_ values were present. As expected, for adult patients, protocol intent C had the greatest dose index values (mean 8.9 ± 2.8 mGy), while B had the lowest one (mean 2.0 ± 0.9 mGy). Protocol intent A had an average dose index value of 5.1 ± 1. Dependence between mean CTDI_vol_ values and protocol intent was statistically significant (*p* = 0.002). The resulting plot of the CTDI_vol_ values is shown in Fig. 2. Only in studies classified under protocol intent C and CTDI_vol_ values of acquisitions with and without contrast were stated. The mean CTDI_vol_ obtained with contrast scans was statistically higher than the mean CTDI_vol_ reported for no contrast scans (11.68 mGy vs 7.99 mGy, *p* = 0.009); the plot of Fig. 2 shows these results. In paediatric studies, it was not possible to calculate a mean CTDI_vol_ because this datum was not reported for any of the selected studies. Effective dose (ED) values were not taken into account here due to the difficulty in making a comparison of these data, as they were derived from different calculation methods.

### CT protocol districts used in PET/CT

Table 6 summarizes the corresponding scan district per each CT scan purpose. In purpose B, the *WB acquisition* modality amounted to 46% (12 out of 26), *WB + segmentary CT scans* (pelvis, liver and head and neck) to 11.5% (3 out of 26) and *CT segmentary acquisition scan* to 11.5% (3 out of 26) for heart and 4% (1 out of 26) for chest. Twenty-seven percent (7 out of 26) of papers did not report a specific protocol (N.A.). For purpose A, only *WB CT scans* were reported. In purpose C, *WB CT scans* were 96% (23 out of 24), and 4% (1 out of 24) CT were *segmentary acquisition scans* (head and neck). Protocol D intent amounted to 100% for *WB CT scan for anatomic localization + abdominal CT acquisition for diagnostic purposes*. Thirteen *WB* studies did not report the protocol intent.

**Table 6:** Results of CT protocol intent distributions in PET/CT

Protocol intent	Scan district	No.	X-ray contrast use
A	WB	22	0
B	WB	12	
	WB + pelvis	1	1
	WB + liver	1	
	WB + H&N	1	
	Heart	3	2
	Chest	1	
	N.A.	7	
C	WB	23	5
	Head and neck	1	1
D	WB + Abdomen	2	1
N.A.	WB	13	1
Total		87	11

*A* CT acquisition for anatomic localization and attenuation correction, *B* CT acquisition for attenuation correction only (ultra-low dose), *C* CT acquisition for diagnostic purposes and attenuation correction, *D* WB CT acquisition for anatomic localization and attenuation correction + CT segmentary acquisition for diagnostic purposes, *N.A.* data not available

In Table 6, it is worth noting that x-ray contrast is employed prevalently in protocols with diagnostic purposes. In particular, for protocol intent C, the x-ray contrast was used in 25% of the studies (6 out of 24), while for protocol intent D, this percentage grew at 50% but only because 2 studies were present. For protocol intent B, the contrast medium was used for heart district scans in 2 cases out of 3 (75%); and for the *WB + pelvis*, it was always used but only because just one study was present.

### CT clinical protocol and dosimetric optimisation

**Fig. 3:**
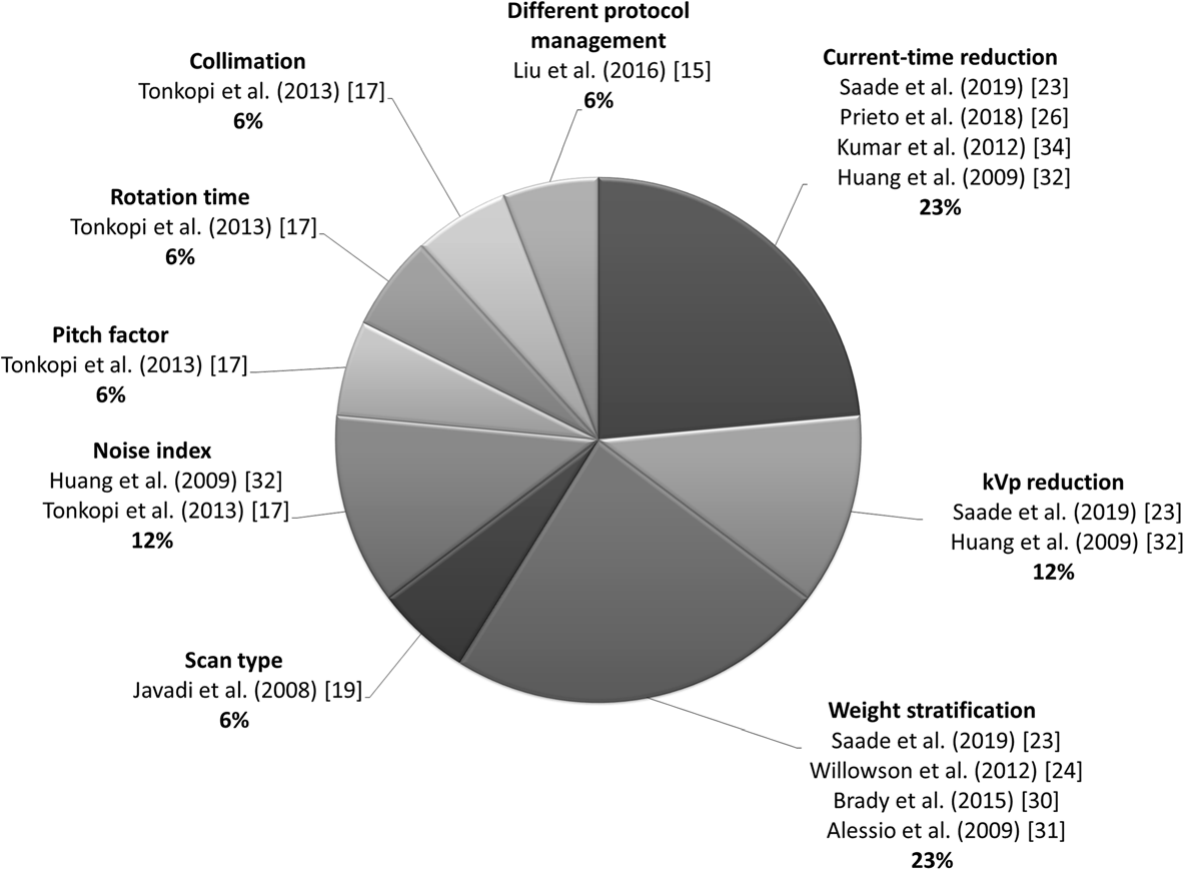
Distribution of the CT optimisation strategies used in PET/CT examination

Table 7 shows the optimisation aim for each clinical protocol intent. Indeed, for protocol intent A, all papers (22 out of 22) revolved around dose optimisation of clinical protocols and in all of the studies WB scans were analysed. In protocol intent B, we found that the clinical protocol was reported in 11 studies (7 *WB*, 1 *segmentary* and 3 *WB + segmentary* acquisitions); on the other hand, dose optimisation protocols were present in 7 out of 25 protocol descriptions (5 *WB*, 2 *segmentary* and 7 *N.A.* acquisitions). For protocol intent C, we found clinical protocols in 2 studies (17 *WB,* 1 *segmentary* and 7 *WB + segmentary* acquisitions). Finally, in protocol intent D, clinical protocols were examined in 2 studies (1 *WB + segmentary* and 1 *WB + segmentary acquisition + three-phase* acquisitions). In 13 out of 87 studies, the optimisation aim was not reported; in 2 papers (*WB*), there was a clinical protocol; and in 11 works, a dose optimisation protocol (*WB*) was applied. As a summary result, we reported in Fig. 3 the distribution of CT optimisation strategies/techniques used in PET/CT examinations.

**Table 7:** Optimisation aim distribution per protocol intent and scan district

Protocol intent	Optimisation aim	Scan district				
		Segmentary	WB	WB + segmentary	WB + segmentary+three-phase	N.A.
A	Y		22			
B	N	1	7	3		
	Y	2	5			7
C	N	1	17	7		
D	Y			1	1	
N.A.	N		2			
	Y		11			
Total		87				

*A* CT acquisition for anatomic localization and attenuation correction*, B* CT acquisition for attenuation correction only (ultra-low dose), *C* CT acquisition for diagnostic purposes and attenuation correction, *D* WB CT acquisition for anatomic localization and attenuation correction + CT segmentary acquisition for diagnostic purpose, *N.A.* data not available, *N* clinical protocol description only, *Y* clinical dose optimisation protocol

## Discussion

This systematic review focused on the description of different applications of CT scans in PET/CT exams; it included 24 articles for a total of 2948 patients and two papers containing large multicentre studies for comparing the results [7, 35] as shown in Fig. 1. The first paper used for the comparison of our results was made by Fahey et al. [7] and focused their work on the dosimetric aspects of paediatric PET/CT exams. The second work, conducted by Jallow et al. [35], described diagnostic reference levels of CT scans in WB PET/CT studies.

Fahey et al. [7] argued that no consistent guidelines are available for CT acquisitions in PET/CT, especially when a paediatric PET/CT exam was involved; in addition to this, they discussed the variability of this study protocol using 19 North American paediatric institutions. They limited their analysis to GE and Siemen scanners. As a general rule, the automatic selection of tube current modulation was used in the majority of the protocols, both for diagnostic and non-diagnostic intents. When the automatic kV selection was available, it was principally used for diagnostic intents. It is worth noting their description of Boston Children’s Hospital approach; through studying a multiseries protocol in 5 different scenarios, they estimated that the effective dose range for a 10-year-old patient was 0.79–3.09 mSv. They concluded that merging diagnostic and non-diagnostic acquisitions into a single multiseries scan can reduce the dose up to 44%. Moreover, they reported an estimated effective dose for diagnostic and non-diagnostic CT for several ages (1-, 5-, 10-, 15-year-old and medium adult patient). For adult protocols, they estimated about 4.8 mSv for diagnostic intents and 1 mSv for non-diagnostic ones, while for 15-, 10-, 5- and 1-year-old patients, the doses employed for the two intents, respectively, ranged between 2–0.6 mSv, 2.1–0.8 mSv and 1.2–0.6 mSv. Their results seem to be lower than those reported by Alessio et al. [31] for paediatric patients, but the limitation of effective dose usage due to different patient dimensions has to be taken into account. In fact, Alessio et al. described a series of CT protocols based on patient weight as reported in Table 3.

Jallow et al. [35] studied the CT acquisition parameters used during PET/CT examinations from 154 sites between 2010 and 2014. They estimated CTDI_vol_ using ImPACT (Imaging Performance Assessment of CT scanners defined by NHS England) CTDI dosimetry tables. Distributions of CTDI_vol_ values were generated, and they found out that mean CTDI_vol_ varied between 6.8 mGy and 7.5 mGy, although the 75th percentile ranged between 9.7 mGy and 10.2 mGy. A limitation of Jallow’s study is that CTDI_vol_ values were not correlated to protocol intents; however, their results are comparable with those obtained in this paper shown in Table 2.

In our review, we focused our attention on the study of CT protocols within the various protocol intents (Table 5). This could be of interest because of the limited number of works investigating this topic. In adult patients, the variability of CT protocols is strictly correlated with protocol intent as shown in Fig. 2; however, in paediatric patients, the protocol variability depended on both the protocol intent and patient size. Regarding this latter issue, only Alessio et al. [31] described the dependence between weight ranges and effective doses; in their case, just protocol intent A was investigated, and no CTDI values were reported. Furthermore, effective doses (EDs) were investigated on phantoms and not on a cohort of paediatric patients except for Alessio et al. [31] and Sonoda et al. [13].

The optimisation of a paediatric protocol CT is a very important issue (due to the higher sensitivity to radiation). In a standard radiology examination, the optimisation can be performed on a wide range of parameters, e.g. selective organ shielding, number of phases, collimation, pitch (increasing it can result in a shorter scan time and in a dose reduction), FOV, kVp (dose halves when lowering it from 120 to 80 kVp), mA-tube rotation time product (it is linearly dependent on dose) and radiation dose modulation techniques [36]. Conversely, in a PET/CT context, it is not always possible to operate on all the acquisition parameters and dose saving strategies as it is not useful given the PET acquisition duration. A common strategy to address this problem is to create more personalized protocols mainly based on patient weight, while for adult patients there is less stratification of protocols. In addition to this, the reduced size of the young patient is the main reason for the less absolute variation of acquisition parameters in paediatric protocols.

Other than protocol intents, we have also addressed the specific protocols required for each purpose. WB scan protocols still remain the most studied (Table 6). So far, some district-specific protocols are present and used by themselves, while in some cases, their combination was described. In addition, each combined protocol was studied in correlation with the x-ray contrast agent use. The contrast medium is more used for diagnostic CT intents and in multiseries CT protocols in combination with high-dose segmentary scans. However, it is also used in scans employed only for attenuation correction. Brix et al. [16] described their experience with iodine contrast agent used in PET/CT scans in four hospitals. They reported the experience of four centres: the first two used separate low-dose CT scans acquired for attenuation correction in addition to a contrast-enhanced CT scan, while the other two institutes used a single contrast-enhanced CT scan for both diagnostic evaluation and attenuation correction. They observed that contrast medium use led to severe artefacts in the attenuation correction process, since anatomical structures with a high attenuation coefficient in the CT scan (corresponding to an increase in Hounsfield Units) may be confounded with bones by the attenuation correction algorithm, as a result of an over-evaluation of local attenuation coefficients. Nevertheless, Beyer et al. [37] demonstrated that the artefacts of the chest veins visible on the scan of the attenuation correction and caused by the administration of the intravenous contrast medium can be reduced through the use of an ad hoc protocol that changes the direction of PET acquisition from caudocranial to craniocaudal and applies a delay time of 50 s (longer than the previous one which instead provided 30 s).

Son et al. [18] mentioned a dedicated head and neck CT protocol for recurrent and metastatic lesions in post-surgical differentiated thyroid carcinoma patients in routine applications. They used a diagnostic protocol (with iodine contrast medium, if necessary) from the cranial top to the thoracic inlet when a patient did not have a previous diagnostic CT exam within 4 weeks before the exam. The same CT diagnostic acquisition was used also for attenuation correction.

Liu et al. [15] reported a specific enhanced CT protocol to study hepatocellular carcinoma; they experienced an increased patient dose when adding a triple-phase contrast-enhanced abdomen scan. They justified the dose increase with an improved risk to benefit ratio.

Eiber et al. [25] described the use of a specific CT protocol for restaging biochemical recurrent prostate cancer. They used an enhanced diagnostic WB CT in portal phase followed by a segmentary low-dose chest CT.

Javadi et al. [19] showed a first experience of iodine-enhanced coronary morphology and physiology study, comparing a low-dose gated step and shoot scan with a helical low-dose CT. Step and shoot acquisition reduced patient doses without causing losses in image quality.

Goldberg et al. [12] reported the use of PET/CT to predict the response of rectal tumors after 1 week of preoperative radiochemotherapy. They used an oral and an intravenous contrast medium to perform CT scans focusing on the pelvis district.

Except for Son et al. [18], other authors did not explicitly indicate if the contrast-enhanced CT substituted the radiological one. This information could be very interesting for protocol dosimetric optimisation.

One limitation of our study is the lack of data regarding the type of tracer used in the PET study; we assumed that this information does not influence the choice of CT acquisition parameters.

Based on our results, CT protocol intent is a great source of patient dose variability; thus, it should be carefully defined by physicians, while planning personalized diagnostic pathways. The most used strategies to reduce CT dose are current-time reduction and weight-based stratification as shown in Fig. 3. In paediatric patients, the use of weight-based acquisition protocols justifies the variability of parameters, together with protocol intents. Four articles suggested a weight-based method to optimise the CT protocol [23, 24, 30, 31], two papers proposed a method based on the variation of the tube current [26, 34] and three studies compared different protocol acquisition techniques [15, 19, 32]. Indeed, Tonkopi et al. [17] presented the comparison between two CT protocols in terms of noise index (from 25 to 27.1), pitch (from 1.75 to 1.35), rotation time (from 0.8 s to 0.5 s) and beam collimation (from 16 × 0.625 to 16 × 1.25). As a result, they optimised the CT acquisition, reducing average CTDI_vol_ from 6.4 to 4.3 mGy (min-max range from 1.7–10.7 mGy to 1.5–7.1 mGy) without compromising diagnostic image quality. They evaluated 11 anatomical structures using a qualitative metric (4-point scale) focusing on overall quality, noise, contrast resolution and edge definition.

In the context of diagnostic uses of CT scans, we hope that more studies will be carried out rigorously to identify a specific pathology for which the PET/CT study can be considered the first diagnostic investigation (thus, avoiding an additional diagnostic CT scan).

Some PET/CT studies that may require multiseries acquisitions with segmentary CT or angio/CT need careful and clear clinical evaluation and justification.

Several other questions remain to be addressed about strategies for optimising CT patient doses, e.g. considering new CT acquisition protocols and including the use of iterative algorithms. On the other hand, those methods are very difficult to compare, due to their implementation technologies which are different for every manufacturer. In some cases, the iterative selection influences the acquisition parameters; in other cases (or other manufacturers), the acquisition phase is independent of the reconstruction one. In this latter case, the dose saving should be previously planned. Thus, in this review, it is quite difficult to describe how these different algorithms work. However, it could be an interesting issue and it should be addressed in a future multicenter study.

Only Brady et al. [30] investigated the use of a CT iterative algorithm (ASiR, GE Healthcare, Waukesha, WI) to minimize the noise in ultra-low-dose CT scans for the attenuation correction purpose. They investigated the impact of radiation-reduced CT acquisition parameters, reconstructed with ASiR. This study was assessed on phantom (Catphan 700, The Phantom Laboratory, Salem, NY). Then, the findings were used to address the effects of a protocol optimisation in a cohort of 140 paediatric and young adult patients. The effective dose saving estimates were in a range of 62–86% depending on the patient characteristics.

An interesting approach could be to correlate a dosimetric index not only to the different CT purposes (i.e. anatomic localization, attenuation correction and diagnostic) but also to the clinical purpose (i.e. oncology, infection/inflammation, brain, cardiac and bone). Recently, Bebbington et al. [38] described a survey of CT doses in hybrid PET/CT and SPECT/CT examinations where they suggested new national diagnostic reference levels (NDRLs).

## Conclusion

This review provides an overview of the state of the art about CT protocol uses within PET/CT scans in the light of limited guidance and literature about this topic.

In our opinion, a preliminary correct protocol intent identification by the physician is necessary to optimise patient doses. According to this review, there is a lack of scientific data and consistent guidelines about CT protocol optimisation in PET/CT exams. Also, the dose optimisation in PET/CT exams depends heavily on the correct implementation of the CT protocol. In addition to this, considering the latest technology advances (i.e. iterative algorithms development), we suggest a periodic quality control audit to stay updated on new clinical utility modalities and to achieve a shared standardisation of clinical protocols.

In conclusion, this study pointed out the necessity to better identify the specific CT protocol use within PET/CT scans, taking into account the continuous development of new technologies.

## Data Availability

All data generated or analysed during this study are included in this published article.
